# Strategies and Techniques for Ileourethral Approximation During Robotic Intracorporeal Neobladder Reconstruction: An International Survey and Video Collection, in Collaboration with the European Association of Urology Robotic Urology Section Scientific Working Group

**DOI:** 10.1016/j.euros.2025.03.018

**Published:** 2025-05-05

**Authors:** Maria Chiara Sighinolfi, Simone Assumma, Peter Wiklund, Richard Gaston, Andrea Minervini, Alex Mottrie, Reza Mehrazin, Abdullah Erdem Canda, Enrico Panio, Filippo Gavi, Luca Sarchi, Tommaso Calcagnile, Filippo Turri, Fabrizio Di Maida, Luca Lambertini, Hubert Jhon, Rafael Coelho, Abolfazi Hosseini, Kris Maes, Anup Kumar, Sudhir Kumar Rawal, T.B. Yuvaraja, Giovanni Enrico Cacciamani, Antonio Celia, Costantino Leonardo, Francesco Greco, Roberto Falabella, Filippo Annino, Antonio Galfano, Angelo Porreca, Alessandro Crestani, Paolo Umari, Riccardo Bertolo, Alessandro Antonelli, Rocco Papalia, Ugo Falagario, Riccardo Schiavina, John Sfakianos, Neerja Tillu, Carl Wijburg, Vincenzo Tondolo, Sergio Alfieri, Alberto Breda, Giorgia Gaia, Stefano Terzoni, Marcio Covas Moschovas, Vipul Patel, Bernardo Rocco

**Affiliations:** a Fondazione Policlinico A. Gemelli IRCSS, Rome, Italy; b The Mount Sinai Hospital, New York, NY, USA; c Università degli Studi di Firenze, Florence, Italy; d OLV Hospital, Aalst, Belgium; e Koç University, Istanbul, Turkey; f ASST Santi Paolo e Carlo, Milan, Italy; g ASST Lariana, Como, Italy; h KSW Kantonsspital Winterthur, Winterthur, Switzerland; i Hospital Sírio-Libanês, Sao Paulo, Brazil; j Karolinska Institutet, Stockholm, Sweden; k Hospital da Luz, Lisbon, Portugal; l Vardhman Mahavir Medical College and Safdarjung Hospital, New Delhi, India; m Rajiv Gandhi Cancer Institute and Research Centre, New Delhi, India; n Kokilaben Dhirubhai Ambani Hospital, Mumbai, India; o University of Southern California, Los Angeles, CA, USA; p Ospedale San Bassiano, Bassano del Grappa, Italy; q Università degli Studi di Roma La Sapienza, Rome, Italy; r Policlinico San Pietro, Ponte San Pietro, Italy; s Azienda Ospedaliera Regionale San Carlo, Potenza, Italy; t Ospedale San Donato, Arezzo, Italy; u ASST Grande Ospedale Metropolitano Niguarda, Milan, Italy; v Istituto Oncologico Veneto IRCCS, Padova, Italy; w Ospedale Santa Maria della Misericordia, Perugia, Italy; x St George’s University Hospitals NHS Foundation Trust, London, UK; y Università degli Studi di Verona, Verona, Italy; z Università Campus Bio-Medico di Roma, Rome, Italy; aa Università degli Studi di Foggia, Foggia, Italy; bb Università degli Studi di Bologna, Bologna, Italy; cc Rijnstate Hospital, Arnhem, The Netherlands; dd Fundació Puigvert, Barcelona, Spain; ee AdventHealt Global Robotic Institute, Celebration, USA; ff Hospital Saint Augustin, Bordeaux, France

**Keywords:** Bladder cancer, Robotic cystectomy, Intracorporeal neobladder, Bowel, Anastomosis, Surgical technique

## Abstract

**Background and objective:**

The use of robotic-assisted radical cystectomy (RARC) with intracorporeal urinary diversion has increased rapidly in the past decade. The approximation of the ileum toward the urethral stump could be a demanding step. Whereas the techniques for reconstruction have been described in detail, a comprehensive depiction of strategies to facilitate neobladder-urethral approximation is lacking. This manuscript and video collection provide a summary of the techniques and maneuvers suggested by RARC surgeons.

**Methods and surgical procedure:**

This is a cross-sectional study in collaboration with the European Association of Urology Robotic Urology Section (ERUS) Scientific Working Group that evaluates strategies for ileourethral approximation and anastomosis from surgeons performing RARC with an intracorporeal neobladder. To this purpose, a survey was developed by a single institution with input from experts. The survey included questions on caseload, types of diversions, ileal approximation, and techniques and strategies for overcoming challenges in an ileourethral anastomosis. Responders were recruited among experts from scientific societies and were asked to rate the importance of these tricks on a Likert scale. A video collection was developed thereafter.

**Key findings and limitations:**

Twenty-one surgeons were involved, with five of them having an individual caseload of >300 cases. The Studer (*n* = 9) and Bordeaux (*n* = 9) reconstructions were most used; four operators declared the use of more than one type of diversion. Ileourethral approximation is considered a demanding part of intracorporeal neobladder reconstruction for 86% of participant surgeons. It is perceived as difficult in approximately one out of four surgical cases. Ten surgeons reported at least one conversion to ileal conduit due to impossible ileal descent. The posterior reconstruction was ranked as a useful trick to aid in an ileourethral anastomosis for ten surgeons (48%); a reduction in the Trendelenburg position by nine (43%), the use of small incisions in the mesentery was useful for six (29%) and opening the ileal segment before the anastomosis for five (24%) surgeons.

**Conclusions:**

Some strategies and techniques are available to facilitate ileal descent toward the pelvis to achieve a tension-free ileourethral anastomosis. The knowledge and application of these tricks are important to cope with this demanding step and make intracorporeal neobladder reconstruction easier and safer.

**Patient summary:**

The robotic realization of a neobladder through an intracorporeal approach could be demanding. The associated video presents some surgical strategies to make this step easier and safer, to ensure the achievement of a tension-free neobladder-urethral anastomosis.

## Take Home Message

Neobladder reconstruction can be performed using various approaches, and standardization of the procedure is crucial for optimal outcomes. Techniques and maneuvers are available to facilitate the ileal descent toward the pelvis, ensuring a tension-free ileourethral anastomosis. Understanding and application of these techniques—individually or in combination—are essential for managing this challenging step.

## 1 Introduction

Radical cystectomy (RC) is the treatment of choice, potentially with perioperative chemotherapy, for muscle-invasive bladder cancer and some high-risk non–muscle-invasive bladder cancer cases [1,2]. However, RC is impacted by high rates of complications. Robotic-assisted RC (RARC) emerged in 2003, and its use has grown steadily due to its potential to reduce surgical complications and improve patient outcomes. RARC combines the advantages of minimally invasive surgery with the enhanced vision and precision of robotic technology with improved dexterity [3]. Extracorporeal urinary diversion (ECUD) was the first approach that has been used to restore the urinary system; however, despite adequate oncological outcomes, the hybrid approach seemingly mitigates clinical results and benefits from RARC with ECUD [[4], [5], [6]].

As surgical experience increases and refinements continue, the approach has shifted gradually from an external to a fully internal technique for urinary diversion [7]; over the past decade, the use of intracorporeal urinary diversion (ICUD) has increased, especially in high-volume hospitals [7,8]. Total intracorporeal RARC with ICUD offers advantages over extracorporeal diversion, including smaller incisions, less pain, and reduced risk of complications due to the absence of open exposure of the bowel [9,10].

Nevertheless, the realization of a neobladder with a full intracorporeal approach could be demanding: detubularization and folding of the bowel inside small spaces, and the need for composite sutures are the main drivers of this complexity [11]. Various techniques for neobladder reconstruction have been developed, some mirroring the steps of open surgery and others adapted specifically for the robotic platform. A recent review article by Piramide et al [11] pointed out nine different techniques reported in the literature: the Studer, Hautmann, Y-shaped, U-shaped, Bordeaux, Pyramid, Shell, Florence Robotic Intracorporeal Neobladder (Florin), and Padua Ileal Bladder (PIB) reconstructions. Each reservoir is characterized by a typical folding technique, and, in general, as the folding number increases, so does the perceived complexity of the reconstruction.

The approximation of the ileum toward the urethral stump is recognized to be demanding as well. Mobilization of the ileum could be affected adversely by adhesions and short or fatty mesentery [12]; a certain degree of Trendelenburg may also hinder ileal descent, and the tension injure the ileourethral anastomosis and provoke direct disruption or ischemic damage, resulting in tissue loss. Some tips and tricks can be used to overcome these issues. Whereas the techniques for reconstruction have been described in detail, a comprehensive depiction of strategies to facilitate neobladder-urethral approximation is lacking.

This manuscript provides a summary of the techniques and maneuvers suggested by RARC surgeons. Exponents mostly of two dedicated scientific societies developed and completed a comprehensive survey.

## 2 Materials and methods

This is a cross-sectional study evaluating tips and tricks for bowel reconfiguration reported by surgeons performing RARC with an intracorporeal neobladder. For this purpose, a survey was developed by Two authors (B.R. and M.C.S.), who drafted the survey based on personal experience and consultations among experts from scientific working groups, which occurred prior to the survey drafting (during congresses and meetings). Surgeons participating in the survey were recruited among the members of the European Association of Urology Robotic Urology Section (ERUS) and of the AGILE group—Italian Group for advanced laparoendoscopic and robotic urological surgery. The inclusion criteria were the execution of RARC at their center and their involvement as surgeons. Different backgrounds (academic/not academic, private, and public) and different levels of individual and center volumes were allowed. The study was carried out in collaboration with the ERUS Scientific Working Group.

### 2.1 Survey

After a section collecting surgeon baseline demographics, the survey consisted of general questions addressing the annual caseload of and approaches to RARC, and personal opinion to overcome a difficult approximation of the ileum toward the urethral stump. Expert opinion was required for some proposed tricks, which were rated with a 5-point Likert scale evaluating the importance of each trick (0 = very unimportant; 5 = very important). A summary of the survey is herein reported.

Questions:

1. How many RCs (open, laparoscopic, or robotic) do you perform in 1 yr?
2. How many RARC do you perform in 1 yr?
3. How many RCs with neobladder reconstruction have you performed in your career?
4. Considering robotic RC (RARC) only, which types of diversions do you perform?
5. If you perform robotic neobladders, which type are these?
6. Do you perform intracorporeal or extracorporeal neobladder reconstruction?
7. How many degrees of Trendelenburg do you apply in the case of neobladder reconstruction?
8. Considering RARC only, how often do you perform this kind of diversion in 1 yr? (Neobladders)
9. In your opinion, what are the most challenging steps of a robotic cystectomy and why?
10. In your opinion, what is the most challenging step of neobladder reconstruction and why?
11. How often do you perceive that ileal approximation to the urethral stump is challenging?
12. How often have you failed ileal approximation to the urethral stump in your career? (Approximately)
13. In the case of difficult approximation, what “tricks” do you deem useful? (Reduce the Trendelenburg degree)
14. In difficult approximation, what “tricks” do you deem useful? (Small incisions in the ileal mesentery to increase the length)
15. In the case of difficult approximation, what “tricks” do you deem useful? (Use of vessel loops around the ileum to facilitate traction down to the pelvis)
16. In the case of difficult approximation, what “tricks” do you deem useful? (Posterior reconstruction)
17. In difficult approximation, what “tricks” do you deem useful? (Opening of the ileal segment before an ileourethral anastomosis)
18. In the case of difficult approximation, what “tricks” do you deem useful? (Assistance from the bedside operator)
19. As far as posterior reconstruction is concerned, which kind of reconstruction do you use to decrease anastomotic tension? That is, single stitch, single layer, double layer, etc.
20. As a final question, do you have any other remarks or suggestions about this topic?

### 2.2 Participants and methodology of the survey

Participants were recruited voluntarily without any incentive; they were told upfront about the length of the survey, and the principal investigators (M.C.S. and B.R.) were clearly named together with their contacts. The survey was built using Google Forms and sent to participants via mail. All results were collected in Google Sheets and exported into Excel. The survey was developed according to the Checklist for Reporting Results of Internet E-surveys (CHERRIES), which is part of the Enhancing the QUAlity and Transparency Of health Research (EQUATOR) network [13]. No personal information or patient-related data were collected; thus, the survey was not password protected. The survey was sent in a single round and was shared only between investigators. Responders could review and change their answers (through an “erase” button). The “view rate” was not available.

### 2.3 Statistics

A statistical analysis was performed with SAS 9 (SAS Inc., Cary, NC, USA). Categorical variables were analyzed as frequencies. The internal consistency of the questionnaire was assessed by the calculation of Cronbach α coefficient. The significance threshold for all calculations was 5%. Categorical variables were analyzed as frequencies and compared with the chi-square test (Fisher’s exact test if expected frequencies in the contingency tables were <5).

## 3 Results

All questionnaires were completed. Cronbach α was satisfactory (0.724), suggesting the reliability of the questionnaire and reproducibility of the answers under similar circumstances.

### 3.1 Surgeons’ characteristics

Twenty-one surgeons were involved, and all of them were males. Four surgeons reported an institutional annual caseload of >100 RCs, regardless of the approach; three reported 51–100 cases; and the remaining surgeons reported <50 cases yearly. As far as the robotic RC annual caseload is concerned, six surgeons perform >50 RARCs each year. When addressing the total number of RARCs with intracorporeal neobladders performed in their career, five surgeons have performed >300 cases so far, three surgeons have performed 100–299 cases, and the remaining surgeons have performed <100 cases. Intracorporeal neobladder reconstruction is the type of diversion performed mostly by 15 surgeons; the remaining used to perform ileal conduit more frequently than a neobladder.

### 3.2 Neobladder reconstruction

The Studer (*n* = 9) and Bordeaux (*n* = 9) reconstructions were the most used diversion type; the Y-shaped reconstruction was used by two, and Florin and Vescica Ileale Padovana were used by two surgeons each. A single surgeon reported using the pitcher pot neobladder. Four operators declared the use of more than one type of orthotopic diversion. The reconstruction was performed intracorporeally in all cases. The degree of the Trendelenburg applied was highly variable and ranged from 0° (single surgeon) to 30°. Twelve surgeons used a Trendelenburg degree ranging from 15° to 20°. Seventeen surgeons (81%) declared to perform posterior reconstruction, regardless of the importance they attribute to this technique.

Nerve sparing (*n* = 8) and pelvic nodal dissection (*n* = 4) are considered the most difficult steps of RARC; ileourethral approximation is considered a demanding part of intracorporeal neobladder reconstruction for 86% of participant surgeons (Table 1). In clinical practice, ileourethral approximation is perceived as difficult in approximately one out of four surgical cases. Ten surgeons reported at least one conversion to ileal conduit due to impossible ileal descent; a single surgeon reported such conversion in up to 30% of cases.

**Table 1:** Challenging steps of RARC and intracorporeal neobladder reconstruction as reported by participants

	*N* (%)
*Perceived challenging steps of RARC*	
Nerve sparing	8 (38)
Pelvic nodal dissection	4 (19)
Management of the bladder volume in case of advanced disease or obesity	3 (14)
Dissection between the bladder and vagina in organ-sparing cases	2 (10)
Intestinal anastomosis because of the angle of the entering stapler	2 (10)
Management of the DVC	1 (5)
*Perceived challenging steps of intracorporeal neobladder reconstruction*	
Ileourethral approximation for a tension-free anastomosis	18 (86)
Folding (symmetry and number of folding)	2 (10)
Choice of proper ileal length	1 (5)

DVC = dorsal venous complex; RARC = robotic-assisted radical cystectomy.

Posterior reconstruction was ranked as a useful trick (Likert scale >3) to aid in an ileourethral anastomosis for ten surgeons (48%); a reduction in the Trendelenburg position for nine, the use of small incisions in the mesentery was useful for six and opening the ileal segment before the anastomosis for five surgeons. The assistance from the bedside operator was considered useful for eight console surgeons. Table 2 summarizes the results considering how important these tricks were rated >50% on the Likert scale.

**Table 2:** Strategies and techniques to enhance ileourethral approximation and rating of importance

In case of difficult approximation, what tricks do you deem useful?	Important (defined as Likert >3), *n* (%)
Technique/surgical strategy	
Posterior reconstruction	10/21 (48)
Incision of the mesentery	6/21 (29)
Opening the ileal segment	5/21 (24)
Maneuvers	
Reducing the Trendelenburg	9/21 (43)
Aid of the assistant	8/21 (38)
Use of vessel loops around the mesentery	1/21 (5)

AS reported by 81% of surveyed surgeons, posterior reconstruction was performed in a double-layer fashion by nine surgeons, in a single-layer fashion by seven surgeons, and with a three-layer technique by a single surgeon.

Other tricks include using an inflated Foley catheter that is retracted by the assistant to push down the neobladder, as suggested by three authors. As final open comments, four authors remarked on the importance of standardizing the procedure to achieve the best surgical and functional outcomes.

## 4 Discussion

The descent of the ileum and its approximation toward the urethral stump could be challenging in some cases, as reported by 86% of the surgeons participating in this survey. Currently, the intestinal segment mostly used to reconstruct a neobladder is the terminal ileum. Owing to its size and stretchiness, the ileum is extendible and may hold more urine at lower pressure, limiting kidney damage and metabolic issues.

The mesentery holds the ileum and is responsible for its mobilization during surgery. During embryogenesis, within the middle and lower region of the mesentery, the developing intestine progressively lengthens, curves, and coils, and the mesentery follows this growth accordingly. The ileocecal region of the mesentery contains a significant volume of lymphatic tissue, and folds and switches and continues to lengthen throughout the human life course [14,15].

Short and fatty mesentery may adversely impact the descent of the ileal segment. Their length and tightness are connected to the ability to mobilize the ileum to reach the pelvis. Another anatomical factor likely to contribute to bowel elongation is the ileum's width, as Annino et al [16] described in an investigational setting. The ileum is a cylinder the hemicircumference of which is prone to interindividual variability. The authors found that variations of a few millimeters of the ileal width may determine variations in the volume of the reconfigured neobladder. Similarly, it could be speculated that width—as hemicircumference—may also impact the ability of the ileum to stretch further and reach distant sites such as the urethral stump [16].

According to the current study, ten out of 21 surgeons reported some cases of failure to achieve neobladder reconstruction due to this occurrence. Based on this surgical issue, we collected tips and tricks from experts to facilitate this step. The tips and tricks may be divided into (1) surgical strategies/techniques and (2) maneuvers that reduce the distance and support ileourethral approximation.

Among surgical techniques, posterior reconstruction is one of the most performed (81% of the participant surgeons) techniques and is recommended by the half as a useful trick to face a challenging ileourethral anastomosis. It begins once the ileum is approximated to the urethra and has been described fully in 2021 [17,18]. Similar to the one originally described for radical prostatectomy [17], it connects the residual Denonvillier’s fascia to the rhabdosphincter in a first layer, and then it connects the posterior aspect of the tubularized bowel segment to the posterior part of the urethral stump. In this original technique, it is performed with a double-armed barbed suture that is finally tied with a progressive tightening to avoid suture cutting through the urethral stump. The intervention proceeds with the realization of a 1.5-cm incision on the bowel, close to the posterior reconstruction, which represents the novel bladder neck, which is finally anastomosed to the urethral stump without tension, given the support of the prior posterior approximation.

The first description of posterior reconstruction was performed on 11 male patients who underwent a Studer reconfiguration [18]. Beyond technical advantages, the authors reported daytime and nighttime continence rates of 100% and 44%, respectively, at 12 mo. Rocco et al [19] further described the same step during a Bordeaux reconfiguration and reported the requirement of a negligible time (5 min, range of 4–8 min in 35 patients). The authors concluded that the posterior reconstruction is simple and reproducible, and willing to maximize the approximation of the bowel and reduce the tension on the anastomosis. Some participant surgeons reported performing the step differently, for example, using a single suture without a double-layer fashion.

Other surgical strategies include the use of small incisions in the ileal mesentery. The trick has been rated as important by 29% of participants and was first described by Collins et al [12] in 2014. Incisions should be superficial and done in a line parallel to the ileum to avoid damage to the mesenteric vessels below [12]. In some cases, the ileal segment fails to reach the urethral stump properly: opening of the ileal segment may be useful to elongate it, even if the distance to be reached should be limited to a few millimeters. The lysis of adhesions—especially at the cecum level—may help mobilize the ileum further [12].

Maneuvers to reduce the distance between the ileum and the urethra include reducing the Trendelenburg position, which 43% of the participant surgeons considered important. Remarkably, the degree applied for an intracorporeal neobladder is highly variable among participants, ranging from 0° to 30°. Another trick is application of external perineal pressure to make the urethral stump closer to the chosen segment.

The aid of the assistant in manipulating the bowel is important for 38% of surgeons. By assisting in a laparoscopic fashion, the bedside operator can feel the traction applied to each segment and can thus choose the tract that best fits the descent. Second, for some surgeons, the assistant is responsible for the lower retraction of the chosen segment on the right side, whereas the fourth arm provides traction on the left side with the robotic grasper. The maneuver can be facilitated by using two vessel loops that are passed around the intestine through the mesentery at each side of the part that will be anastomosed to the urethra. The vessel loops have elastic properties and may be stretched without the risk of damaging healthy tissue; these avoid a direct grip of the grasper on the bowel, performed by either the assistant or the grasper [12].

Other surgeon participants reported scattered tricks, such as using a Foley catheter inflated or anchored to the neobladder to push down the ileum toward the urethra, while performing the anastomosis to decrease the tension or reduction of the pneumoperitoneum during the anastomosis. Another suggestion borrowed from general surgery for the management of ulcerative colitis involves preparing the second-to-last ileal segment to form an optimal pouch that can extend low into the pelvis, reaching the anal level. This is accomplished by transecting the second-to-last mesenteric vessel, enabling the ileum to descend further to the anal margin for pouch construction. This technical adjustment can lengthen the mesentery effectively, facilitating a tension-free anastomosis.

This study is not devoid of limitations. First, the survey reports opinions from urologists involved in scientific societies but may have missed other surgeons performing RARC with an intracorporeal neobladder. The survey methodology was used to strengthen the importance of each step and allowed us to describe them in a video collection. Second, the opinion and results of experts may not be reproducible in centers with surgeons having less expertise. Third, the survey collects experts’ opinions but fails to provide clinical outcomes of each surgical technique/trick. The lack of measurable outcomes and metrics may be considered a limitation too. However, we should remark that the descent of the bowel is often the result of a combination of the reported tricks; thus, attributing the success to a single maneuver may be difficult.

To our knowledge, this is the first article collecting strategies to help in overcoming a difficult ileourethral approximation and anastomosis. The awareness of these solutions, together with the best coordination between the console and the assistant surgeon, are key elements to make this challenging step easier, to create a tension-free anastomosis, and to maximize the outcomes of neobladder reconstruction.

## 5 Conclusions

There are different ways to perform neobladder reconstruction, and the procedure's standardization is a key element. Some maneuvers and techniques are available to facilitate ileal descent toward the pelvis to realize a tension-free ileourethral anastomosis. The knowledge and application of these tricks, alone or in combination, are important to cope with this demanding step and make intracorporeal neobladder reconstruction easier and safer.

  ***Author contributions*:** Maria Chiara Sighinolfi had full access to all the data in the study and takes responsibility for the integrity of the data and the accuracy of the data analysis.

  *Study concept and design*: Sighinolfi, Rocco, Wiklund, Gaston, Minervini, Mottrie, Mehrazin, Canda.

*Acquisition of data*: Calcagnile, Panio, Assumma.

*Analysis and interpretation of data*: Gavi, Cacciamani, Bertolo.

*Drafting of the manuscript*: Sighinolfi, Assumma, Panio, Rocco, Gavi.

*Critical revision of the manuscript for important intellectual content*: Rocco.

*Statistical analysis*: Gavi, Cacciamani, Bertolo.

*Obtaining funding*: None.

*Administrative, technical, or material support*: None.

*Supervision*: Sarchi, Calcagnile, Turri, Di Maida, Lambertini, Jhon, Coelho, Hosseini, Maes, Kumar, Rawal, Yuvaraja, Cacciamani, Celia, Leonardo, Greco, Falabella, Annino, Galfano, Porreca, Crestani, Umari, Bertolo, Antonelli, Papalia, Falagario, Schiavina, Sfakianos, Tillu, Wijburg, Tondolo, Alfieri, Breda, Gaia, Terzoni, Moschovas, Patel.

*Other*: None.

  ***Financial disclosures:*** Maria Chiara Sighinolfi certifies that all conflicts of interest, including specific financial interests and relationships and affiliations relevant to the subject matter or materials discussed in the manuscript (eg, employment/affiliation, grants or funding, consultancies, honoraria, stock ownership or options, expert testimony, royalties, or patents filed, received, or pending), are the following: None.

  ***Funding/Support and role of the sponsor*:** None.
